# Rise of the beta-lactams: a retrospective, comparative cohort of oral beta-lactam antibiotics as step-down therapy for hospitalized adults with acute pyelonephritis

**DOI:** 10.1017/ash.2024.70

**Published:** 2024-07-26

**Authors:** Athena L.V. Hobbs, Vagish S. Hemmige, Katie L. Reel, Theresa C. Jaso, Dusten T. Rose, Katherine M. Shea

**Affiliations:** 1 Cardinal Health Innovative Delivery Solutions, Stafford, TX, USA; 2 Department of Medicine, Montefiore Medical Center, Bronx, NY, USA; 3 Department of Pharmacy, Sentara Healthcare, Virginia Beach, VA, USA; 4 Department of Pharmacy, Ascension Seton Medical Center, Austin, TX, USA; 5 Department of Pharmacy, Ascension Dell Seton Medical Center-UT, Austin, TX, USA

## Abstract

**Objective:**

The aim of this study was to determine if oral beta-lactam therapy is non-inferior to alternative therapy at discharge following inpatient treatment with an IV cephalosporin for acute pyelonephritis.

**Design:**

Institutional Review Board (IRB)-approved, multicenter, retrospective, non-inferiority cohort (15% non-inferiority margin).

**Setting:**

Six hospitals within two healthcare systems.

**Patients:**

Hospitalized patients admitted to the medical floor with acute pyelonephritis without urologic abnormalities who received cefazolin or ceftriaxone followed by step-down therapy.

**Methods:**

Patients were discharged with either an oral beta-lactam or an oral alternative agent (ie, fluoroquinolone or trimethoprim-sulfamethoxazole) to complete therapy. The primary objective was treatment failure defined as a composite of hospital readmission or an ED visit for a urinary cause within 30 days of discharge of the index hospitalization. Data were extracted manually from the electronic medical record.

**Results:**

A total of 211 patients were included; 122 received an oral beta-lactam and 89 received an oral alternative agent at discharge. There was no difference in 30-day treatment failure between the two groups (4.9% vs 5.6% for oral beta-lactams vs oral alternatives, respectively. Absolute difference = 0.7%; 95% CI -5.4% to 6.8%; *P* = .82). The median length of hospital stay, number of patients treated with intravenous ceftriaxone, duration of IV therapy, and median duration of oral therapy were no different between groups.

**Conclusions:**

In non-ICU patients admitted for pyelonephritis without urologic abnormalities, oral beta-lactams were non-inferior to oral alternatives for step-down therapy. In finding non-inferiority between the regimens, we show the feasibility of administering oral beta-lactams to complete therapy for acute pyelonephritis.

## Introduction

Patients hospitalized for pyelonephritis traditionally receive intravenous antimicrobial therapy with transition to guideline-recommended non-beta-lactam oral therapies (eg, fluoroquinolone, trimethoprim/sulfamethoxazole [TMP/SMX]).^1^ However, due to increasing antimicrobial resistance (AMR) to primary recommended oral therapies and the Food and Drug Administration (FDA) boxed warnings for fluoroquinolones, there is heightened interest in the utility of oral beta-lactams as step-down therapy for treatment of pyelonephritis requiring hospitalization.^2^ Additionally, de-escalation and intravenous-to-oral conversion via step-down therapy are important antimicrobial stewardship interventions to optimize antibiotic prescribing.^3^ Application of antimicrobial stewardship in the management of urinary tract infections (UTIs) including pyelonephritis, can help mitigate the effects of AMR.

The 2010 Infectious Diseases Society of America (IDSA) and European Society for Microbiology and Infectious Diseases (ESCMID) guideline for acute uncomplicated UTIs and pyelonephritis recommends oral agents (ie, fluoroquinolones, TMP/SMX) for patients not requiring hospital admission.^1^ Empiric therapy with fluoroquinolones should be avoided in areas where local AMR rates are >10% to fluoroquinolones, yet North American susceptibility data indicates that *E.coli* exhibit 71.8% and 71.5% susceptibility to levofloxacin and ciprofloxacin, respectively.^4^ Likewise, TMP/SMX is recommended only in areas where local AMR rates are <20% or if the uropathogen is known to be susceptible; however, the susceptibility rate for TMP/SMX is only 76.7% in North America.^5^ IDSA/ESCMID guidelines caution that oral beta-lactams are less effective for the treatment of pyelonephritis, and recommendations regarding oral step-down antibiotic therapy for hospitalized patients are lacking.

Investigators sought to assess the impact of oral step-down beta-lactam therapy compared to an alternative oral agent (fluoroquinolone or TMP/SMX) in patients with acute pyelonephritis requiring hospitalization.

## Patients and methods

### Study design

This multicenter, retrospective, non-inferiority cohort included hospitalized patients with acute pyelonephritis in six hospitals (one academic medical center and five community hospitals) within two healthcare systems who received an IV cephalosporin (ie, ceftriaxone or cefazolin) followed by step-down therapy with either an oral beta-lactam or an oral alternative agent (ie, fluoroquinolone or TMP/SMX). A local inpatient UTI clinical pathway was implemented in all but one community hospital in May 2009. The clinical pathway provided oral antibiotic treatment options for UTIs based on isolated organism(s). For *Escherichia coli*, *Klebsiella* spp., and *Proteus* spp., recommended oral agents included amoxicillin, cephalexin, amoxicillin/clavulanate, TMP/SMX, and fluoroquinolones based on susceptibility of the isolated organism(s).

ICD-9 codes (590.8, 590.9, 590.10, 590.11) were used to identify patients with an admission diagnosis of acute pyelonephritis between January 1, 2014 and December 31, 2016. This study was approved by the institutional review boards of both healthcare systems. The primary outcome was treatment failure defined as a composite of hospital readmission or an ED visit for a urinary cause within 30 days of discharge of the index hospitalization. Urinary cause was defined as anything relating to the genitourinary system. Secondary outcomes included 90-day treatment failure as well as 30- and 90-day hospital readmission and ED visits for any reason.

### Data collection

Demographic, infectious etiology, treatment, and outcomes clinical data were obtained from the inpatient electronic medical record. All data were extracted into a standardized form through manual chart review by four co-authors. Charlson Comorbidity Index data were also collected at baseline to evaluate and compare comorbid conditions between the groups. Baseline is defined as the first value documented in the electronic medical record upon initial admission unless otherwise specified.

In the event that a data point was not available in the patient’s chart, it was assumed that the patient did not meet criteria for said disease state captured in the Charlson Comorbidity Index. Temperature (minimum and maximum when more than one was available within a 24-hour time period) was recorded at baseline and within every 24-hour time period up to 5 days. Data for any hospital readmission or ED visit were collected throughout the Ascension Seton Health Alliance and the Baptist Memorial Healthcare Corporation to account for visits at any facility within those healthcare systems. Hospital readmission data for patients in the Austin, Texas area were also gathered through iCare, a local readmission documentation collaboration between hospitals in the Austin, Texas area to account for hospital readmissions at local hospitals outside the Ascension Seton Health Alliance.

### Study patients

Patients were included if they were between the ages of 18 and 89 years, had an admission diagnosis of acute pyelonephritis, and received at least 24 hours of an intravenous (IV) cephalosporin (ceftriaxone or cefazolin) followed by step-down therapy with either an oral beta-lactam or an oral alternative agent (ciprofloxacin, levofloxacin, or TMP/SMX). Of note, inpatient antibiotic duration was calculated as anticipated duration of systemic concentrations of active drug (eg, 72 hours if patient received three doses of ceftriaxone).

Patients were excluded if they were pregnant; were admitted to the intensive care unit (ICU); received more than one dose of another individual IV antibiotic with Gram-negative activity prior to receiving the IV cephalosporin; received a discharge antibiotic other than an oral beta-lactam, fluoroquinolone, or TMP/SMX; were transferred from an outside hospital when records were not available; had another source of infection (other than bacteremia due to urinary source); or either did not have a baseline fever (temperature >100.3°F) or elevated serum white blood cell (WBC) count (WBC ≥12 X10^9^ cells/L) plus urinary symptoms recorded. Patients were also excluded if they had a urological abnormality that is known to increase the risk of treatment failure including urethral strictures, renal stents, congenital abnormalities, renal cysts, neurogenic bladder, renal calculi, vesicoureteral reflux, renal abscess, or a suprapubic catheter.

### Statistical analysis

An *a priori* one-sided test of non-inferiority revealed that 89 patients were required in each group to achieve 80% power with a non-inferiority margin of 15% and assuming a cure rate of 85% as reported in previous literature.^6^ Patients were collected in a reverse chronological order until power was met in both groups. Beta-lactams were considered non-inferior if the 95% confidence interval margin did not cross −15%. Categorical data were analyzed using Chi-squared or Fisher’s exact test, and continuous data were analyzed using Wilcoxon rank sum or T-test as appropriate. A multivariate logistic regression analysis was performed in a secondary analysis using the primary outcome as the dependent variable with potential confounders included as independent variables. R statistical software was used to conduct all statistical analyses.^7^

## Results

A total of 211 patients were included in the study; 122 received an oral beta-lactam and 89 received an oral alternative agent at discharge. Baseline characteristics were similar between groups other than mean age and female sex (Table 1). Patients were predominantly young females with fewer comorbidities. Additionally, the median duration of IV cephalosporins was roughly 3 days in both groups with a median duration of 7–10 days of oral antibiotics prescribed at discharge.

**Table 1.** Demographic, clinical, and treatment characteristics

	PO BL (n = 122)	PO Alternative (n = 89)	*P* value
Male, n (%)	5 (4.1)	11 (12.4)	.44
Female, n (%)	117 (95.9)	78 (87.6)	.03
Age, years	44 (±18)	40 (±17)	.045
Median weight, kg (IQR)	72.0 (62.0, 85.5)	70.0 (60.0, 81.8)	.34
Healthcare System, n (%)			
Ascension Seton Health Alliance	113 (92.6)	77 (86.5)	.14
Baptist Memorial Healthcare Corporation	9 (7.4)	12 (13.5)	
CT or US suggestive of pyelonephritis during admission, *n* (%)	70/90 (77.8)	56/74 (75.7)	.75
Blood culture positive, *n* (%)	7/82 (8.5%)	9/82 (11.0%)	.60
Baseline WBC × 10^3^/mcL	14.4 (±5.2)	13.6 (±4.9)	.35
Baseline temperature, °F	100.9 (±1.96)	101.1 (±1.74)	.59
Baseline heart rate, beats/min	105.6 (±24.8)	105.9 (±19.0)	.83
Baseline systolic blood pressure, mm/Hg	123.7 (± 20.3)	125.3 (± 20.5)	.65
Charlson comorbidity index			
0, *n* (%)	72 (59.0)	55 (61.8)	.13
1, *n* (%)	33 (27.1)	29 (32.6)	
2+, *n* (%)	17 (13.9)	5 (5.6)	
Receipt of ceftriaxone, *n* (%)	56 (45.9)	53 (59.6)	.05
Receipt of cefazolin, *n* (%)	66 (54.1)	36 (40.4)	.05
Median IV duration, days (IQR)	3.0 (2.1, 4.0)	2.8 (2.0, 4.0)	.27
Median oral antibiotic duration, days (IQR)	7.0 (7.0, 10.0)	10 (7.0, 10.3)	.08
Median Inpatient length of stay, days (IQR)	2.7 (2.0, 3.5)	2.6 (1.8, 3.6)	.48

Results are presented as mean ± standard deviation unless otherwise specified. Baseline temperature is the maximum temperature within 24 hours of initiation of antibiotics. The first value in the first 24-hour period from admission was used for other baseline vitals. IV antibiotic was either cefazolin or ceftriaxone.

IQR, interquartile range; CT, computed tomography; US, ultrasound; WBC, white blood cell; °F conversion to °C = (temperature in °F – 32) * 5/9.

**Figure 1.**
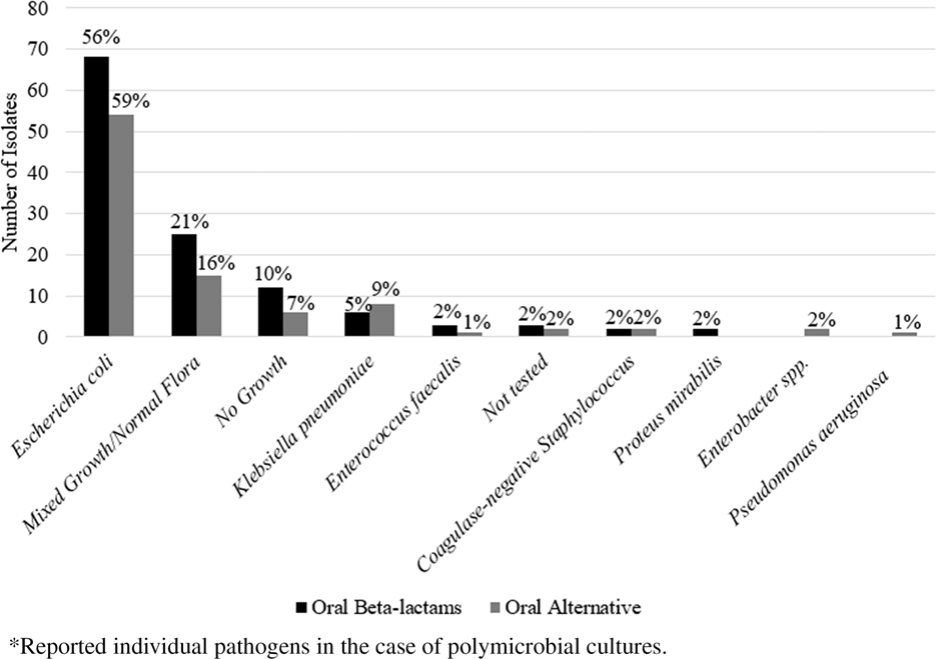
Urinary isolates.

**Figure 2.**
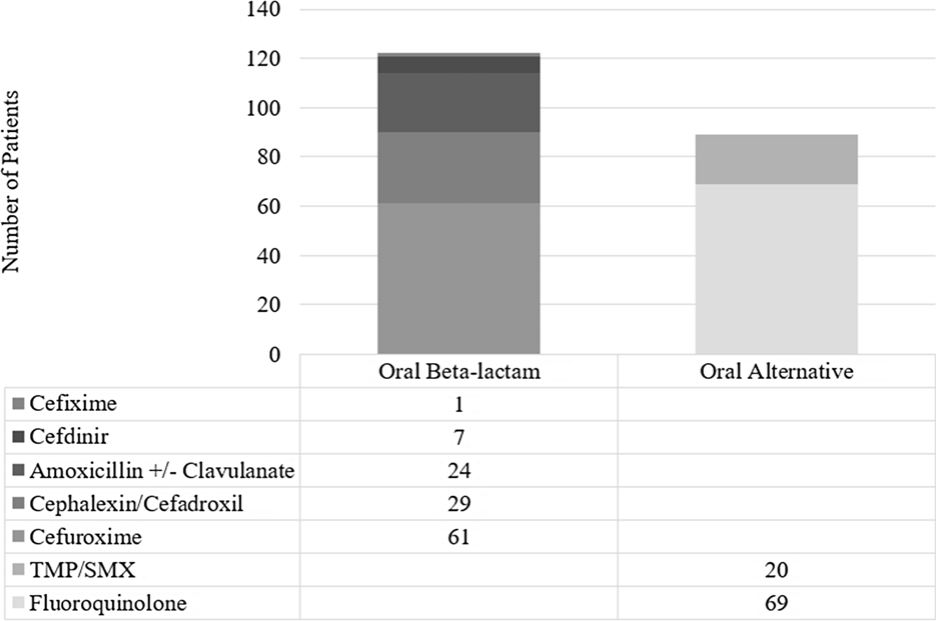
Discharge oral antibiotic choice.

The urinary pathogens isolated in each group consisted primarily of *E. coli* (58%) and are depicted fully in Figure 1. Notably, only five patients did not have a urine culture performed. 50% of patients who received an oral beta-lactam at discharge received cefuroxime, followed by cephalexin/cefadroxil (24%), and amoxicillin +/- clavulanate (19%). 78% of patients who received an alternative oral antibiotic received a fluoroquinolone at discharge (Figure 2).

There was no difference in 30-day treatment failure when comparing the two groups (4.9% vs 5.6% for oral beta-lactams vs. oral alternatives, respectively. Absolute difference = 0.7%; 95% CI −5.4% to 6.8%; *P* = .82; Table 2). There was also no difference in 90-day treatment failure due to a urinary cause when comparing patients discharged on oral beta-lactams vs oral alternatives. Likewise, there was no difference between groups when evaluating either 30- or 90-day all-cause treatment failure. These findings persisted when potential confounders were controlled for in the multivariate logistic regression (Table 3).

**Table 2.** Outcomes

	Oral Beta-lactam, n (%) (n = 122)	Oral Alternative, n (%) (n = 89)	Risk difference (95% CI)	*P* value
30-day treatment failure (urinary cause)	6 (4.9)	5 (5.6)	−0.7% (−5.7%, 8.2%)	.82
30-day all-cause treatment failure	12 (9.8)	12 (13.5)	3.6% (−6.0, 13.9%)	.41
90-day treatment failure (urinary cause)	7 (5.7)	6 (6.7)	−1.0% (−6.5%, 9.3%)	.76
90-day all-cause treatment failure	18 (14.8)	19 (21.3)	6.6% (−4.4%, 19.2%)	.21

*Treatment failure is defined as a composite of hospital readmission or ED visit.

**Table 3.** Adjusted outcomes using multivariate logistic regression

Adjustment variable	30-day treatment failure (urinary cause)		30-day all-cause treatment failure		90-day treatment failure (urinary cause)		90-day all-cause treatment failure	
	OR (95% CI)	*P* value	OR (95% CI)	*P* value	OR (95% CI)	*P* value	OR (95% CI)	*P* value
None	0.87 (0.26–2.94)	.821	0.70 (0.30–1.64)	.412	0.84 (0.27–2.60)	.765	0.64 (0.31–1.30)	.216
CCI	0.89* (0.26–3.02)	.847	0.68* (0.28–1.62)	.389	0.86* (0.28–2.68)	.801	0.66* (0.32–1.38)	.274
Age (linear)	1.02 (0.30–3.52)	.975	0.74 (0.31–1.75)	.492	0.92 (0.30–2.88)	.892	0.66 (0.32–1.36)	.263
Age (categorical)	1.03 (0.30–3.59)	.957	0.77 (0.32–1.82)	.551	0.95 (0.30–2.97)	.926	0.70 (0.34–1.45)	.335
Ceftriaxone	0.98 (0.29–3.37)		0.72 (0.31–1.71)	.463	0.93 (0.30–2.91)	.902	0.64 (0.31–1.32)	.228
Male gender	0.90* (0.27–3.06)	.870	0.71 (0.30–1.67)	.436	0.88* (0.28–2.71)	.817	0.65 (0.32–1.34)	.244
Bloodstream infection	1.18* (0.34–4.59)	.790	0.89 (0.37–2.15)	.791	1.18 (0.38–3.66)	.781	0.93 (0.44–1.96)	.840
CT-confirmed pyelonephritis	0.69 (0.17–2.61)	.552	0.72 (0.27–1.90)	.513	0.68 (0.20–2.35)	.543	0.67 (0.29–1.55)	.349
WBC ≥ 17 X10^3^/mcL	0.87 (0.26–2.95)	.823	0.70 (0.30–1.64)	.418	0.87 (0.28–2.68)	.802	0.64 (0.31–1.30)	.213
Baseline temperature ≥ 100.4 (°F)	0.82 (0.24–2.80)	.750	0.69 (0.29–1.62)	.381	0.81 (0.26–2.52)	.722	0.63 (0.31–1.29)	.209
Study site	0.88* (0.24–3.16)	.840	0.81* (0.33–2.02)	.653	0.94* (0.29–3.06)	.915	0.72 (0.34–1.53)	.394

*Perfect predictor for at least one level; at least some observations dropped.

Baseline temperature is the maximum temperature within 24 hours of initiation of antibiotics; CT, computed tomography; WBC, white blood cell; °F conversion to °C = (temperature in °F – 32) * 5/9.

## Discussion

These findings suggest that step-down therapy with oral beta-lactams (eg, cefuroxime, cephalexin, and amoxicillin/clavulanate) for the treatment of hospitalized patients with pyelonephritis provides similar outcomes as oral fluoroquinolones and TMP/SMX. The 2010 IDSA/ESCMID international practice guideline recommendations do not address oral beta-lactam step-down therapy and refer back to the 1999 UTI guidelines that state beta-lactams are not as efficacious for pyelonephritis based on four randomized controlled trials from the 1980s and early 1990s. Two of these studies used oral ampicillin in the beta-lactam arm, another only evaluated microbiologic response, and the last compared a short beta-lactam duration of therapy.^8–11^ In addition to all of these being very small studies, they were plagued by significant limitations including the low bioavailability of oral ampicillin, the reliance on microbiologic cure alone instead of also evaluating clinical cure, and a shorter than required duration of therapy for beta-lactams. Interestingly, when presented at IDWeek 2023, the draft IDSA clinical practice guidelines for the treatment of complicated urinary tract infections, including pyelonephritis, recommend conversion to oral therapy (including beta-lactams with good urinary excretion at highest labeled dose) in patients with clinical improvement, hemodynamic stability, and source control.^12^

Published literature specifically evaluating oral step-down therapy for the treatment of pyelonephritis is limited to three small, prospective, randomized studies. When comparing IV ceftriaxone (n = 41) vs oral cefditoren (n = 41) as step-down therapy after 3 days of IV ceftriaxone, there was no difference in clinical cure 95.1% vs 100%, respectively (p=0.15).^13^ Similarly, when comparing IV ceftriaxone (n = 54) vs oral cefixime (n = 51) after receiving 24 hours of IV ceftriaxone, rates of clinical cure were similar between the two groups 91% vs 92%, respectively (*P* = 1).^14^ Finally, when comparing oral ceftibuten (n = 79) vs oral norfloxacin (n = 79) after receiving 2–4 days of IV cefuroxime, rates of clinical success were similar 82% vs 92%, respectively (95% CI 0.81–1), though there were fewer microbiologic relapses in the norfloxacin group (11%) versus the ceftibuten group (25%), *P* < 0.05.^15^ The first of these studies defined clinical cure as the absence of dysuria or symptoms of pyelonephritis, while the latter two studies defined clinical cure as resolution of urinary symptoms including flank pain as well as resolution of fever.^11–15^

Although neither specifically assessed use of oral beta-lactams for step-down therapy, two additional retrospective studies found similar outcomes when assessing the use of oral cephalosporins compared to fluoroquinolones and TMP/SMX for treatment of pyelonephritis. A retrospective chart review by Lin and colleagues found no difference in the composite outcome of treatment failure within 30 days between oral cephalosporins and fluoroquinolones (15.3% vs 17.4%). Of note, 92.5% of patients received initial intravenous therapy for 1–2 days.^16^ Likewise, Fosse et al. performed a retrospective observational cohort study and found no significant difference in UTI recurrence at 30 days between use of an oral cephalosporin versus an alternative agent (16% vs 17%; *P* = 0.851). Additionally, treatment with an oral cephalosporin was not identified as a significant risk factor for UTI recurrence (OR = 0.91, 95% CI 0.56–1.50; *P* = 0.722).^17^ These studies provide limited data that the use of oral cephalosporins, typically after initial IV administration, may be an oral option for the treatment of pyelonephritis.

Additionally, four retrospective cohort studies have assessed the impact of oral beta-lactams as step-down therapy for treatment of bacteremia due to a urinary source.^18–21^ Three of the four studies found no significant difference in mortality and/or recurrence between oral beta-lactams and either fluoroquinolones or TMP/SMX after a median of 4–5 days of IV therapy; however, these studies may have been underpowered to detect a difference.^18–20^ Alzaidi and colleagues found a non-significantly higher risk of recurrence (aHR 2.19 [0.95–5.01]) in patients who received highly bioavailable beta-lactams (amoxicillin, amoxicillin/clavulanate, cephalexin) vs non-beta-lactams; however, 70% of these patients were not optimally dosed for the treatment of bacteremia and received only a median of 3 days of IV therapy prior to oral conversion.^21^ These studies demonstrate promise for the use of oral beta-lactams as step-down therapy for bacteremia due to a urinary source. However, due to the lack of reported patients with pyelonephritis in these studies, generalizability to this patient population is limited.

### Strengths/Limitations

This is the largest study comparing clinical outcomes for oral step-down therapy which was also powered to determine a difference in the primary outcome of treatment failure. Based on the available clinical data and anecdotal observations, we explored the effectiveness of oral beta-lactams for step-down therapy in light of high rates of AMR to first-line therapy described in current guidelines.

Patients with urological abnormalities were excluded from this study in an effort to limit confounders outside of antibiotic therapy alone that could influence treatment failure. Like most real-world studies, microbiologic and clinical cure could not be confirmed after patients were discharged. Thus, investigators assessed hospital readmission or ED visits as a surrogate for treatment failure. Though this captures potential 30-day hospital visits, we were unable to ascertain whether patients experienced less severe urinary symptoms that did not require a hospital visit. Furthermore, hospital readmission and ED visit data for one large community hospital in Memphis was limited to the 22 hospitals within that healthcare system. Despite this limitation, the majority of patients (90%) were treated at one of the five hospitals in Austin, Texas where investigators had access to a community-wide collaborative data share and were able to capture readmissions or ED visits to any local hospital in the area. However, due to the nature of this data collection, some patients could have experienced a hospital admission or ED visit outside the included hospitals.

Like most studies evaluating acute pyelonephritis, the majority of patients in this study were young females with limited comorbidities, which may limit the extrapolation of this data to the male and elderly population. Additional limitations to clinical application include patients with urologic abnormalities and immunocompromise as these comorbidities were not assessed. Oral antibiotic susceptibility was also unable to be assessed due to the lack of many oral beta-lactams on commercially available susceptibility panels. The Clinical Laboratory and Standards Institute provides susceptibility interpretation for several oral cephalosporins based on utilizing cefazolin as a surrogate for uncomplicated cystitis; however, recommendations and guidance are lacking regarding susceptibility inference for the treatment of pyelonephritis.^22^ Finally, as a retrospective study, the step-down therapy antibiotic selection, timing, and dosing were at the discretion of the prescriber. We relied on accurate and consistent documentation in the electronic medical record including discharge antibiotic choice, dose, and duration, as we had no way to verify that prescriptions were filled or taken by patients.

### Clinical implications

This is the largest comparative study evaluating clinical outcomes of beta-lactam oral step-down therapy for acute pyelonephritis. In non-ICU patients without urologic abnormalities, oral beta-lactams were non-inferior to an oral alternative for step-down therapy for hospitalized patients with pyelonephritis. Finding non-inferiority between the regimens demonstrates the feasibility of administering oral beta-lactams in light of current AMR as well as FDA-boxed warnings for fluoroquinolones. Investigators hope that this retrospective cohort study provides an opportunity for antimicrobial stewardship optimization in utilizing oral beta-lactams for step-down treatment of pyelonephritis but recognize that randomized, controlled trials are needed to strengthen the level of evidence.

## Research transparency and reproducibility

Databases are not publicly available.
